# Lineage-Specific Associations between the Resistome and Mobilome across 10,500 Globally Distributed *Acinetobacter baumannii* Genomes

**DOI:** 10.34133/csbj.0123

**Published:** 2026-06-09

**Authors:** Thunchanok Yaikhan, Sirikan Suwannasin, Thitaporn Dechathai, Kamonnut Singkhamanan, Sarunyou Chusri, Rattanaruji Pomwised, Monwadee Wonglapsuwan, Komwit Surachat

**Affiliations:** ^1^ Department of Biomedical Sciences and Biomedical Engineering, Faculty of Medicine, Prince of Songkla University, Hat Yai, Songkhla 90110, Thailand.; ^2^ Division of Infectious Diseases, Department of Internal Medicine, Faculty of Medicine, Prince of Songkla University, Hat Yai, Songkhla 90110, Thailand.; ^3^ Division of Biological Science, Faculty of Science, Prince of Songkla University, Hat Yai, Songkhla 90110, Thailand.

## Abstract

**Background:** *Acinetobacter baumannii* is a major nosocomial pathogen with extensive antimicrobial resistance, yet the extent to which resistome burden covaries with mobilome architecture across clonal backgrounds remains unclear at the global scale. Using a curated dataset of 10,500 high-quality genomes, this study examined lineage-resolved relationships among AMRFinder-detected resistance determinants, insertion sequence (IS) burden, plasmid diversity, predicted plasmid mobility, and virulence-associated gene presence. **Results:** The population structure was highly uneven and dominated by a small number of lineages. Using total AMRFinder burden, genome-level associations with IS burden and plasmid replicon diversity were modest (Spearman rho = 0.259 and 0.246, respectively). In a sensitivity analysis using an acquired-like burden metric that excluded OXA-51 family hits, *bla*_ADC_ family hits, predefined intrinsic or conserved chromosomal background loci, and non-antibiotic resistance classes, the median burden decreased from 17 to 9 hits per genome. Under this filter, acquired-like burden remained associated with IS/mobile genetic element burden (rho = 0.237) and showed a slightly stronger association with plasmid replicon diversity (rho = 0.285), with both relationships persisting after excluding genomes from China and the United States. Mobilome features remained markedly heterogeneous across lineages, whereas virulence-associated gene presence profiles were comparatively conserved. **Conclusions:** These findings support a lineage-structured view of resistance-associated genomic variation in *A. baumannii*. However, the observed genome-wide associations were small in magnitude and should be interpreted as patterns of co-occurrence rather than direct evidence of mechanism or causality. Sensitivity analyses suggest that plasmid-related features show a more robust relationship with acquired-like resistance burden than IS counts alone. This distinction is relevant for genomic surveillance, but it should be interpreted together with lineage imbalance, sampling bias, and the limitations of gene-presence-based inference.

## Introduction

*Acinetobacter baumannii* has emerged as one of the most formidable nosocomial pathogens worldwide, particularly in intensive care units and among immunocompromised patient populations. Its remarkable ability to survive under environmental stress, persist on hospital surfaces, and rapidly acquire antimicrobial resistance (AMR) determinants has positioned it among the highest-priority critical pathogens identified by the World Health Organization. Carbapenem-resistant *A. baumannii*, frequently associated with multidrug-resistant phenotypes, represents a major threat to global public health [1–4].

Historically, AMR in *A. baumannii* has been largely attributed to horizontal gene transfer mediated by plasmids, transposons, and other mobile genetic elements (MGEs) [5,6]. The widespread dissemination of OXA-type carbapenemases and metallo-beta-lactamases such as New Delhi metallo-beta-lactamase has reinforced the view that resistance evolution in this species is primarily driven by the acquisition of mobile resistance determinants [7–9]. However, increasing genomic evidence suggests that resistance dissemination is not uniformly distributed across the species population but is frequently concentrated within a limited number of high-risk clonal lineages [10–13]. Among the best-recognized international clones, the Pasteur ST2 lineage corresponding to International Clone II (IC2) has been repeatedly identified as a globally disseminated, high-risk hospital-associated lineage [14–16].

MGEs constitute a central component of the *A. baumannii* mobilome. Insertion sequences (ISs) can activate adjacent resistance genes, promote composite transposon formation, and drive genomic rearrangements, while conjugative and mobilizable plasmids facilitate horizontal gene transfer between bacterial cells [17,18]. Despite their recognized importance, the relative contributions of chromosomal transposition and plasmid-mediated transfer—and how these processes vary among evolutionary lineages—remain poorly characterized at a global scale.

Most previous investigations of resistome or mobilome dynamics in *A. baumannii* have focused on local outbreaks, regional collections, or relatively small genome datasets. Such limitations restrict the ability to detect large-scale evolutionary patterns and lineage-associated genomic structures [19–21]. In addition, resistome and mobilome features are often examined independently rather than within an integrated population-genomic framework.

In this study, we leveraged a curated dataset of 10,500 high-quality RefSeq genomes to perform a comprehensive lineage-resolved analysis of the global *A. baumannii* population. By integrating multilocus sequence typing (MLST), genome similarity analysis, AMR profiling, IS quantification, and plasmid mobility characterization, we systematically examined the population structure and genomic features underlying resistance evolution. Specifically, we evaluated the extent to which AMR burden is structured by clonal lineage, quantified heterogeneity in mobilome composition across evolutionary lineages, and assessed whether quantitative associations between mobilome components and resistome accumulation differ among distinct genetic backgrounds.

Through this integrative genomic framework, we aim to clarify how clonal expansion and MGE dynamics jointly shape the global resistance landscape of *A. baumannii*. Understanding these lineage-dependent evolutionary processes provides a broader perspective on resistance dissemination and may inform genomic surveillance strategies and predictive epidemiological monitoring of this critical pathogen.

## Materials and Methods

### Genome retrieval and quality filtering

#### Genome collection

All publicly available *A. baumannii* genome assemblies were surveyed from the National Center for Biotechnology Information (NCBI) Assembly database using the NCBI Datasets platform (accessed January 2026) [22]. At the time of data collection, a total of 47,558 genome assemblies were available for *A. baumannii*. Among these, 11,900 assemblies were curated RefSeq genomes with standardized RefSeq annotations, whereas the remaining assemblies consisted primarily of GenBank-only submissions, metagenome-assembled genomes (MAGs), or atypical draft assemblies lacking consistent annotation and quality metadata.

To ensure annotation consistency, comparability across genomes, and analytical reproducibility, only RefSeq-annotated assemblies were retained for downstream analyses [23]. Assemblies classified as MAGs or atypical or low-standard assemblies were explicitly excluded from the dataset [24]. Genome sequence files and assembly metadata were retrieved and consolidated into a unified dataset. Assembly statistics, including genome size, GC content, number of contigs, and contig N50/L50 values, were extracted from the RefSeq metadata.

#### Genome quality assessment and filtering

Genome completeness and contamination were estimated using CheckM (v1.1.3) based on lineage-specific marker genes [25]. Genome quality was evaluated using a combination of structural assembly metrics and lineage-based completeness estimates. Assemblies were retained only if they satisfied stringent quality criteria, including a CheckM completeness of at least 95%, a CheckM contamination level not exceeding 5%, a total number of contigs of 500 or fewer, and a contig N50 value of at least 50,000 bp. Genomes failing to meet any of these thresholds or lacking the required quality metadata were excluded from downstream analyses.

### MLST and population structure analysis

#### MLST assignment and clonal-complex determination

MLST was performed on all quality control (QC)-passed *A. baumannii* genomes using FastMLST (v0.0.15), which enables rapid allele calling against the PubMLST database [26]. Genome assemblies in FASTA format were analyzed using both established *A. baumannii* MLST schemes: the Pasteur scheme [27] and the Oxford scheme [28]. MLST profiles were assigned based on exact matches to curated allele definitions provided by PubMLST.

Typing success differed substantially between the 2 schemes in the FastMLST output used for this study. The scheme corresponding to Pasteur ST designations achieved a high numeric typing rate across the QC-passed dataset (97.2%), whereas the Oxford scheme yielded numeric ST assignments for 22.9% of genomes, with the remainder represented by incomplete, novel, or nonnumeric profiles. Accordingly, Pasteur STs were used as the primary interpretable lineage framework for downstream summaries.

Clonal complexes were then defined from the primary-scheme ST profiles using single-locus variant (SLV) relationships derived from the observed allele profiles. Because these clusters were generated from the present dataset rather than taken from a standardized external clonal-complex nomenclature, they are referred to here as SLV-derived clonal complexes (SLV-CCs) and should be interpreted as internal analytic groupings rather than formal epidemiologic clone labels. International clone terminology is used only when referring to recognized ST-level designations such as Pasteur ST2/IC2.

#### Mash-based genome similarity and clustering analysis

The global population was strongly dominated by Pasteur ST2, which accounted for 6,125 genomes (~58%). When grouped by SLV relationships, ST2 and closely related sequence types formed the largest internal SLV-derived clonal complex (SLV-CC1), comprising 7,968 genomes (75.9%). Beyond ST-based lineage assignments, genome-wide similarity was assessed independently of predefined loci using Mash v2.3. Mash sketches [29] were generated from all QC-passed *A. baumannii* genome assemblies using a *k*-mer size of 21 and a sketch size of 10,000, providing an effective balance between sensitivity and computational efficiency for large-scale genome comparison. Only QC-passed genome assemblies were included in Mash analyses.

Genome-wide Mash distances were computed from the combined sketch file using the Mash triangle algorithm [29], which generates a lower-triangular distance matrix optimized for large datasets. The resulting Mash distance matrix was used as an orthogonal, whole-genome measure of relatedness to support the MLST-defined population structure and to identify clusters of closely related genomes that may not be fully resolved by locus-based typing alone.

A neighbor-joining [30] tree was inferred from the Mash distance matrix using RapidNJ [31], enabling scalable tree reconstruction for the full dataset of 10,500 genomes. For computational stability, Mash distance matrices were reformatted and processed using disk-based caching where required. The resulting Mash-based neighbor-joining tree was used for exploratory visualization of genome-wide similarity and as a supporting framework for lineage-resolved comparative analyses, rather than as a high-resolution phylogenetic reconstruction. Mash distances were not used as a formal near-duplicate exclusion filter before downstream statistical analyses.

### AMR gene profiling

AMR determinants were identified using AMRFinderPlus (v3.11.11) with default parameters. Predicted protein sequences derived from RefSeq-annotated genomes were screened against the curated AMRFinderPlus reference database to identify acquired resistance genes together with intrinsic or resistance-associated loci recognized by the database [32]. Hits were classified according to gene family and predicted antimicrobial class.

For each genome, the total number of detected AMRFinderPlus hits was summarized, and resistance profiles were aggregated at the ST and SLV-CC levels. Genomes lacking detected hits were retained and explicitly recorded as zero-hit cases. Because this summary metric includes both acquired determinants and intrinsic chromosomal resistance-associated loci detected by AMRFinderPlus, it is interpreted here as a broad resistance-content measure rather than a direct estimate of recently acquired antibiotic resistance gene (ARG) accumulation. As a sensitivity analysis, we additionally defined an acquired-like burden metric by excluding OXA-51 family hits, *bla*_ADC_ family hits, selected intrinsic/background loci (*abaF*, *adeC*, *amvA*, *nreB*, and *cxpE*), and non-antibiotic resistance classes such as metal- or disinfectant-associated determinants.

### Mobilome analysis and IS detection

MGEs, including ISs and transposable elements, were identified using MEFinder v1.1.2 [33]. Genome assemblies in FASTA format were analyzed using default parameters, and detected IS elements were annotated based on homology to curated IS databases integrated within the MEFinder pipeline. All *A. baumannii* genomes were submitted to MEFinder. No-hit or empty MEFinder outputs were interpreted as zero detected IS/MGE features; 396 genomes fell into this zero-detection category. Therefore, IS/MGE burden summaries were calculated across the full 10,500-genome dataset. The total number of detected IS-associated hits per genome was used here as a descriptive proxy for mobilome expansion rather than a direct measure of transposition rate.

### Plasmid reconstruction, replicon typing, and mobility classification

Plasmid-associated sequences and their mobility potential were characterized using MOB-suite v3.0.3 [34], a computational framework for plasmid reconstruction, typing, and mobility prediction from draft genome assemblies. All QC-passed genome assemblies were analyzed using the MOB-recon module with default parameters to identify and reconstruct putative plasmid sequences from assembled contigs. MOB-recon assigns contigs to plasmid clusters through comparison against curated reference plasmid databases and sequence-derived genomic signatures, allowing plasmid-derived sequences to be distinguished from chromosomal regions in fragmented assemblies.

Reconstructed plasmids were subsequently analyzed using MOB-typer to infer plasmid typing features, including predicted replicon content, relaxase family assignment, and mobility class. Relaxase genes were classified into established MOB families on the basis of homology to curated reference sequences. For each genome, the number of distinct plasmid replicon types detected by MOB-suite was recorded as a descriptive measure of plasmid diversity for lineage-resolved comparisons.

Plasmid mobility was inferred from the presence or absence of canonical conjugation-associated markers, including relaxase genes, mate-pair formation (MPF) systems, and origin-of-transfer sequences. Plasmids encoding both a relaxase gene and a complete MPF system were classified as conjugative, reflecting the predicted capacity for autonomous transfer. Plasmids carrying a relaxase gene and/or an origin-of-transfer sequence but lacking a complete MPF system were classified as mobilizable, consistent with a requirement for transfer functions provided in trans. Plasmids lacking detectable mobility-associated markers were classified as nonmobilizable. Using these assignments, the proportional distribution of conjugative, mobilizable, and nonmobilizable plasmids was summarized for each clonal complex to compare lineage-specific plasmid mobility architectures across the global dataset.

To maintain analytical consistency, downstream plasmid-based analyses were restricted to genomes with complete MOB-suite outputs. The resulting plasmid replicon diversity and predicted mobility profiles were integrated with Pasteur STs, SLV-derived CC assignments, AMRFinderPlus hit counts, and IS burden for comparative and statistical analyses of lineage-resolved mobilome organization.

### Virulence factor profiling

Virulence-associated genes were profiled for all QC-passed genomes using the Virulence Factor Database (VFDB) [35]. Genome assemblies in FASTA format were screened against the VFDB reference database using ABRicate v1.2.0 with default settings (minimum 80% nucleotide identity and 80% coverage). The total number of VFDB-associated genes detected in each genome was recorded as a summary measure of virulence-associated gene presence.

For lineage-resolved comparisons, virulence gene counts were aggregated by Pasteur-based SLV-CC. In addition, the per-gene prevalence within each SLV-CC was calculated as the proportion of genomes in that lineage carrying the corresponding VFDB hit. These results were interpreted as presence/absence summaries and not as direct measures of virulence expression or pathogenic potential.

### Integrated resistome–mobilome analysis

To assess associations between resistance burden and mobilome structure, per-genome outputs from AMRFinderPlus, MEFinder, plasmid replicon profiling, and plasmid mobility classification were merged with Pasteur ST and SLV-CC assignments. Integrated variables included AMRFinderPlus hit count, IS burden, plasmid replicon type count, and virulence gene count where available.

Genome-level associations between AMRFinderPlus hit burden and mobilome-related variables were evaluated across the full dataset. In addition, lineage-stratified analyses were performed separately for major SLV-CCs to determine whether the strength of these associations differed among genetic backgrounds. At the lineage level, median IS burden, mean plasmid replicon diversity, and the proportion of predicted conjugative plasmids were summarized to compare mobilome configurations among clonal complexes.

### Statistical analysis

All statistical analyses were performed in R. Because per-genome feature counts were not assumed to follow a normal distribution, nonparametric methods were used for lineage-level comparisons. Differences in AMRFinderPlus hit counts, IS burden, plasmid replicon diversity, and virulence gene counts among SLV-CCs were assessed using the Kruskal–Wallis test. Where overall differences were significant, pairwise post hoc comparisons were performed using Dunn’s test with false discovery rate correction for multiple testing when reported.

Associations between AMRFinderPlus hit burden and mobilome-related variables were evaluated using Spearman’s rank correlation coefficient. Correlation analyses were performed both across the full dataset and separately within major SLV-CCs. Given the large sample size, emphasis was placed on the effect-size magnitude (rho) together with statistical significance. As additional robustness checks, we repeated genome-level correlations after excluding genomes from China and the United States and fitted lineage-adjusted negative binomial models with acquired-like AMR burden as the response and IS burden, plasmid replicon diversity, and SLV-CC as predictors.

## Results

### Genome dataset curation and quality filtering

**Fig. 1.**
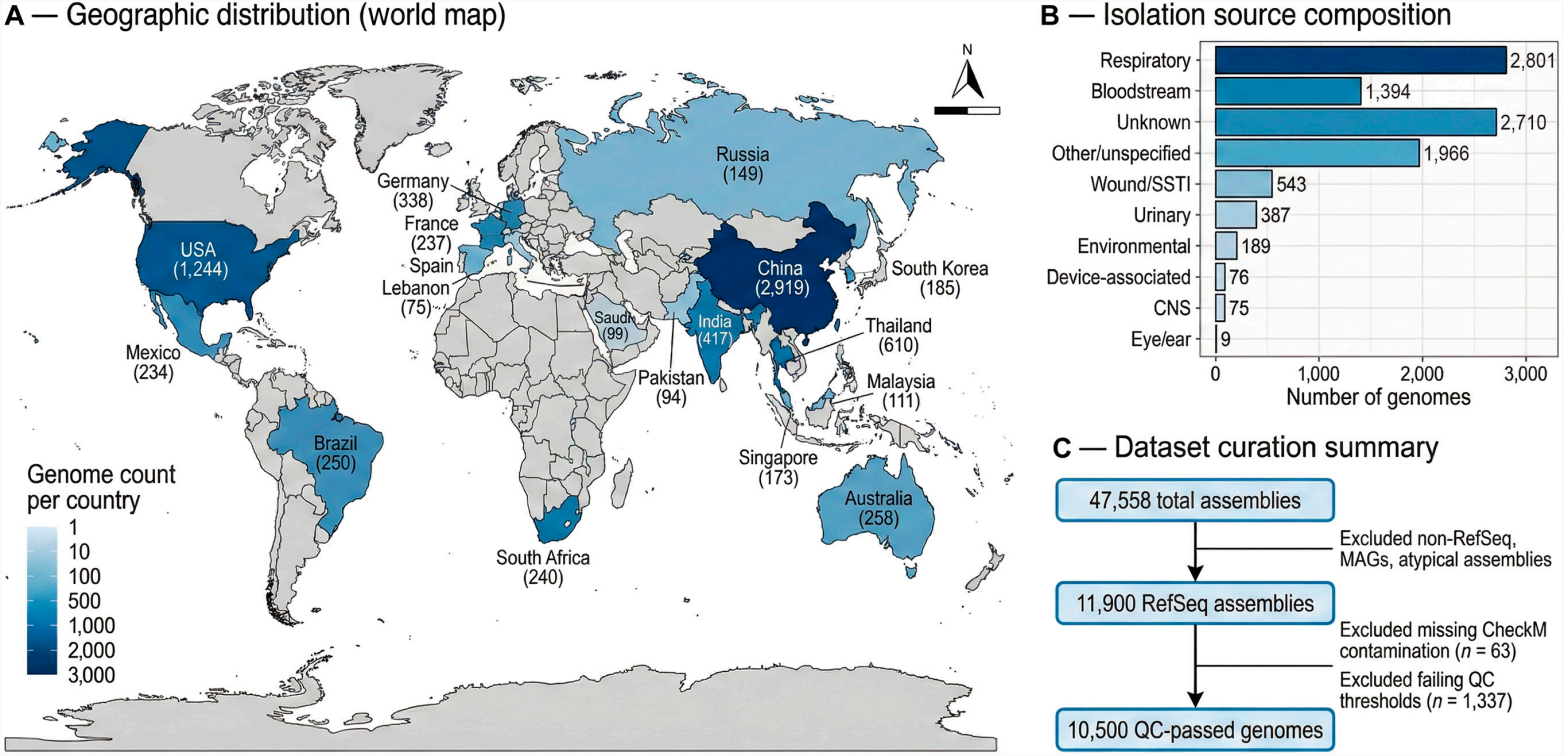
Global distribution, clinical origin, and curation of the *Acinetobacter baumannii* genome dataset. (A) World map showing the geographic distribution of 10,500 high-quality *A. baumannii* genomes by country after normalization of BioSample geographic metadata. Color intensity indicates the number of genomes contributed by each country. (B) Distribution of standardized isolation source categories among the analyzed genomes. Bars represent the number of genomes assigned to each harmonized specimen category. (C) Summary of dataset curation and quality filtering, illustrating reduction from 47,558 publicly available assemblies to 11,900 RefSeq-annotated genomes and final inclusion of 10,500 quality control (QC)-passed genomes retained for downstream analyses.

A total of 47,558 *A. baumannii* genome assemblies were available in the NCBI Assembly database at the time of data collection (2026 Jan 24). Among these, 11,900 assemblies corresponded to curated RefSeq genomes with standardized annotations, whereas the remaining assemblies consisted primarily of GenBank-only submissions, MAGs, or atypical draft assemblies and were therefore excluded to ensure annotation consistency and analytical reproducibility (Fig. 1C). The RefSeq dataset comprised assemblies spanning multiple assembly levels, including 7,681 contig-level assemblies, 3,229 scaffold-level assemblies, 915 complete genomes, and 75 chromosome-level assemblies (Table S3). Following quality filtering, the final dataset retained assemblies across all assembly levels, with the majority remaining at contig or scaffold resolution.

The RefSeq assemblies were subsequently subjected to genome quality assessment using structural assembly metrics and CheckM-based completeness estimates. Genomes were retained only if they satisfied stringent quality thresholds, including a CheckM completeness of at least 95%, a CheckM contamination level not exceeding 5%, a maximum of 500 contigs, and a contig N50 of at least 50 kb. A small subset of assemblies (*n* = 63) lacked CheckM contamination values and were therefore excluded from downstream quality filtering and subsequent analyses (Table S2). After applying these criteria, 10,500 high-quality genomes were retained for all downstream comparative and statistical analyses. Summary statistics of genome quality metrics for the retained dataset are provided in Table S1, and the full Preferred Reporting Items for Systematic Reviews and Meta-Analyses (PRISMA)-based workflow is summarized in Fig. S1 and Table S2.

### Geographic distribution and isolation source composition

After normalization and harmonization of metadata fields, the 10,500 high-quality *A. baumannii* genomes were found to originate from 97 countries (Table S4). The largest contributions were from China (*n* = 2,919) and the United States (*n* = 1,244), which together accounted for 39.6% of the dataset, followed by Thailand (*n* = 610), India (*n* = 417), Germany (*n* = 338), and Australia (*n* = 258). A subset of genomes lacked precise geographic annotation and were classified as unknown (*n* = 940). Collection-year metadata were recoverable for 9,381 genomes (89.3%) and spanned 1900 to 2025, underscoring the cross-sectional and temporally heterogeneous nature of the dataset. Isolation source summaries and metadata harmonization mappings are provided in Tables S5 to S8, and metadata-coverage/bias summaries are provided in Table S28. The global distribution of genomes is visualized in Fig. 1A.

Isolation source metadata were standardized into harmonized specimen labels and broader clinical categories (Fig. 1B and Tables S5 and S6). Most genomes were derived from clinical respiratory specimens, including sputum and tracheal or bronchoalveolar samples (respiratory category, *n* = 2,801), followed by bloodstream isolates (*n* = 1,394), wound and skin/soft tissue samples (*n* = 543), and urinary tract specimens (*n* = 387). Smaller proportions originated from environmental sources (*n* = 189), device-associated specimens (*n* = 76), central nervous system samples (*n* = 75), and eye or ear specimens (*n* = 9). Approximately 2,710 genomes lacked clearly defined isolation source information and were categorized as unknown, while an additional fraction was grouped as other or unspecified clinical materials.

### MLST-based population structure of the QC-passed dataset

**Fig. 2.**
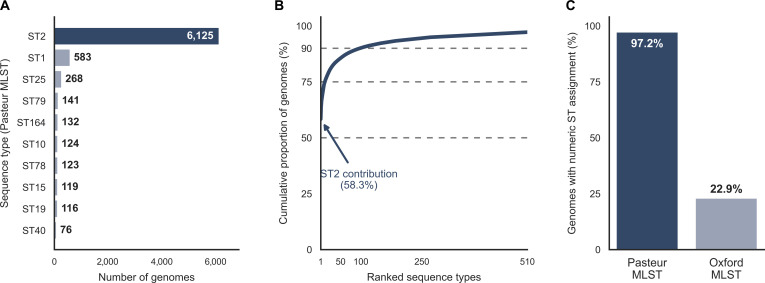
Pasteur multilocus sequence typing (MLST) reveals a highly clonal global population structure of *Acinetobacter baumannii*. (A) Frequency distribution of the most common Pasteur MLST sequence types among the 10,500 quality control (QC)-passed genomes. Bars indicate the number of genomes assigned to each ST. (B) Cumulative abundance curve showing the proportion of genomes represented by ranked Pasteur sequence types across the dataset. (C) Comparison of MLST success rates between the Pasteur and Oxford schemes. Bars indicate the percentage of genomes successfully assigned a numeric sequence type under each scheme.

To resolve population structure across the curated genome collection, we assigned MLST using the 2 commonly used *A. baumannii* MLST schemes (Pasteur and Oxford) (Fig. 2C). MLST assignment was highly scheme dependent: the Pasteur scheme achieved a high typing rate (97.2%), whereas the Oxford scheme yielded numeric ST assignments for 22.9% of genomes, indicating extensive incompleteness or nonnumeric profiles in the Oxford output for this RefSeq-derived dataset. Pasteur MLST revealed a strongly clonal population structure dominated by a small number of epidemic lineages (Fig. 2A and B). ST2 was the most prevalent lineage (*n* = 6,125), accounting for approximately 58% of all QC-passed genomes (6,125/10,500). Detailed ST, country, and SLV-CC summaries are provided in Tables S9 to S13 and Figs. S2 to S5. Because dataset-specific SLV clusters are not equivalent to formal international clone labels, ST2/IC2 terminology is retained at the ST level, whereas the downstream CC labels are used only as internal analytic groupings.

### Clonal-complex structure of the global *A. baumannii* population

Pasteur MLST profiles were grouped into CCs using SLV relationships to organize closely related sequence types into broader internal lineage groupings. The QC-passed dataset showed a highly uneven distribution of these SLV-derived clonal complexes. SLV-CC1 was the dominant grouping, comprising 7,968 genomes (75.9%). This cluster contained Pasteur ST2 and closely related SLVs and therefore captured much of the globally disseminated ST2/IC2 background. These internal groups are referred to consistently as SLV-CCs to distinguish them from formal global clone or epidemiologic clonal-complex nomenclature. Dominant observed Pasteur STs and representative accessions for the major SLV-CCs are provided in Table S29; these should be interpreted as representative observed genotypes rather than phylogenetically inferred ancestral states.

The remaining genomes were distributed across multiple smaller SLV-CCs. The most common among these were SLV-CC4 (*n* = 287; 2.73%), SLV-CC5 (*n* = 187; 1.78%), SLV-CC6 (*n* = 165; 1.57%), SLV-CC13 (*n* = 131; 1.25%), and SLV-CC14 (*n* = 127; 1.21%). Additional genomes were distributed among several low-frequency SLV-CCs and singleton sequence types (Table S12 and Fig. S5). Subsequent analyses were performed using both ST and internal SLV-CC assignments.

### Global resistome architecture and lineage-resolved enrichment

**Fig. 3.**
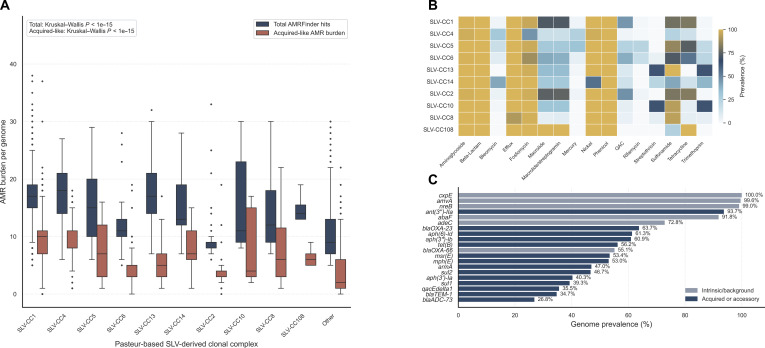
Resistome burden and class-level resistance profiles across global *Acinetobacter baumannii* lineages. (A) Distribution of total AMRFinderPlus hit counts and acquired-like antimicrobial resistance (AMR) burden per genome across Pasteur-based single-locus variant (SLV)-derived clonal complexes (SLV-CCs) among 10,500 quality control (QC)-passed genomes. Boxplots show median and interquartile range (IQR), with whiskers representing 1.5 × IQR; reported *P* values denote overall Kruskal–Wallis tests. (B) Heatmap showing the prevalence of antimicrobial resistance classes across SLV-CCs. Values represent the percentage of genomes within each SLV-CC carrying at least one gene associated with the indicated resistance class. (C) Prevalence of the most frequently detected AMRFinderPlus genes across the full genome collection. Bars indicate genome prevalence and distinguish intrinsic/background loci from acquired or accessory resistance genes.

Genome-wide screening of AMRFinderPlus determinants across the 10,500 QC-passed genomes showed significant variation in total hit burden among SLV-CCs (Kruskal–Wallis test, *P* < 1 × 10^−15^) (Fig. 3A). Resistance class prevalence also varied across lineages, although this burden metric includes both intrinsic/chromosomal resistance-associated loci and acquired genes. Beta-lactam and aminoglycoside-associated hits were prevalent across most major SLV-CCs, whereas several other classes showed more heterogeneous lineage distributions (Fig. 3B). Genome-level AMR burden, class-level prevalence, and top-gene summaries are provided in Tables S14 to S16 and Figs. S6 and S7. Because this metric mixes intrinsic and acquired signals, we also evaluated an acquired-like burden metric. Under this filter, the median per-genome burden decreased from 17 total AMRFinder hits to 9 acquired-like hits (Table S25).

Across the full dataset, several AMRFinderPlus loci were highly prevalent. Intrinsic or chromosomally encoded resistance-associated loci, including efflux-related genes such as *cxpE*, *emrA*, *nreB*, and *abaF*, were detected in nearly all genomes. Among acquired resistance genes, commonly detected loci included *bla*_OXA-23_, *bla*_TEM-1_, *aph(3′)-VIa*, and *aph(6)-Id* (Fig. 3C). These results indicate that the total AMRFinder burden mixes conserved background determinants with acquired ARGs and should therefore be interpreted cautiously.

### Lineage-specific distribution of carbapenemase genes

**Fig. 4.**
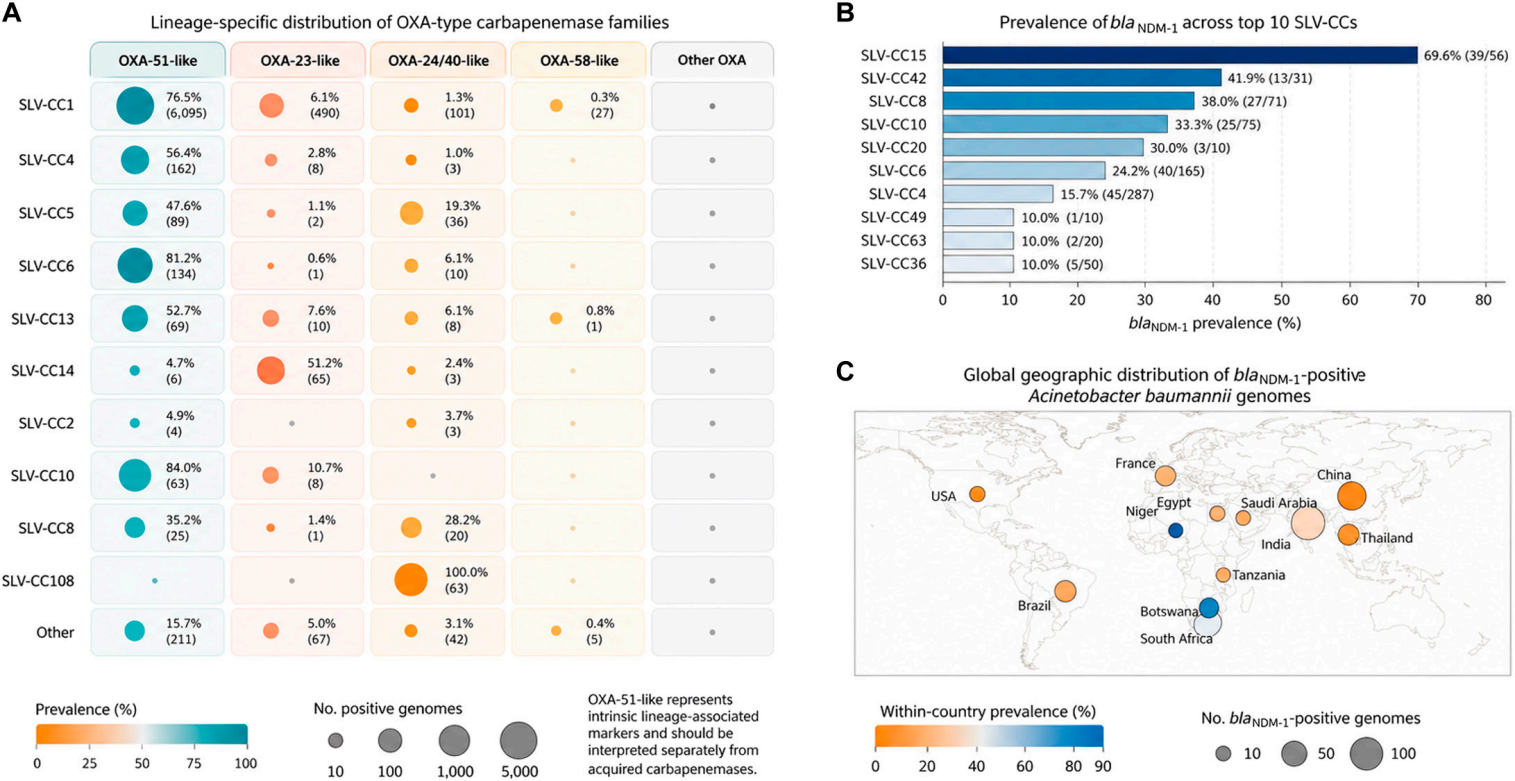
Lineage-specific distribution and geographic dissemination of carbapenemase genes in *Acinetobacter baumannii*. (A) Dot heatmap showing the distribution of OXA-type beta-lactamase families across major Pasteur-based single-locus variant (SLV)-derived clonal complexes (SLV-CCs). Color intensity and circle size represent within-lineage prevalence. OXA-51-like genes are shown as intrinsic lineage-associated markers and should be interpreted separately from acquired carbapenemases. (B) Enrichment of *bla*_NDM-1_ across SLV-CCs with at least 10 genomes, ranked by within-lineage prevalence. (C) Geographic distribution of countries with the largest numbers of *bla*_NDM-1_-positive genomes. Circle size corresponds to the number of *bla*_NDM-1_-positive genomes, and color intensity reflects within-country prevalence among represented genomes.

Carbapenemase genes were widely detected across the global *A. baumannii* dataset. Two major groups were identified: OXA-type class D beta-lactamases and the metallo-beta-lactamase gene *bla*_NDM-1_. Multiple OXA families were observed, including the intrinsic OXA-51-like family and acquired families such as OXA-23-like, OXA-24/40-like, and OXA-58-like enzymes. OXA-51-like genes were detected in nearly all genomes in most SLV-CCs and are interpreted here as intrinsic lineage markers rather than as acquired carbapenemases. In contrast, acquired OXA families showed variable prevalence across lineages, with some SLV-CCs enriched for specific families (Fig. 4A, Table S17, and Fig. S9). For example, SLV-CC1 showed a high proportion of genomes carrying OXA-23-like carbapenemases, whereas other SLV-CCs contained higher proportions of OXA-24/40-like or OXA-58-like enzymes.

The metallo-beta-lactamase gene *bla*_NDM-1_ was detected in 669 of 10,500 genomes (6.37%). Its distribution varied among internal SLV-CCs. SLV-CC1 contained the largest number of *bla*_NDM-1_-positive genomes due to its large population size, although the prevalence within this lineage was 5.40%. Higher within-lineage prevalence was observed in several smaller SLV-CCs, including SLV-CC15 (69.64%; 39/56), SLV-CC42 (41.94%; 13/31), SLV-CC8 (38.03%; 27/71), SLV-CC10 (33.33%; 25/75), and SLV-CC6 (24.24%; 40/165) (Fig. 4B and Table S18). Geographically, *bla*_NDM-1_-positive genomes were identified across multiple regions, with the largest numbers observed in India, South Africa, China, France, Thailand, Brazil, Botswana, Egypt, the United States, and Saudi Arabia after excluding missing or not applicable country labels (Fig. 4C and Fig. S8).

### Mobilome diversity across lineages

**Fig. 5.**
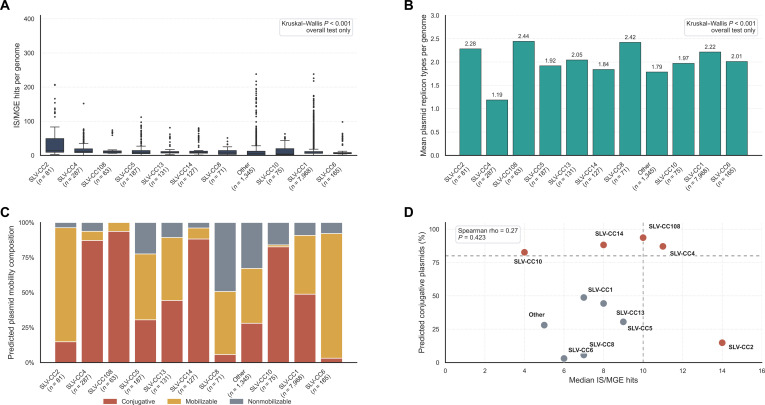
Lineage-structured mobilome heterogeneity across global *Acinetobacter baumannii* lineages. (A) Distribution of insertion sequence (IS)/mobile genetic element (MGE) hits per genome across major single-locus variant (SLV)-derived clonal complexes (SLV-CCs) among all 10,500 genomes. No-hit or empty MEFinder outputs were coded as zero detected IS/MGE features. Boxplots show median and interquartile range. The reported *P* value denotes the overall Kruskal–Wallis test; pairwise Dunn results are provided in Table S20. (B) Mean number of plasmid replicon types per genome across SLV-CCs. Bars represent the mean number of replicon types detected within each lineage. (C) Predicted plasmid mobility composition across SLV-CCs. Stacked bars represent the proportion of conjugative, mobilizable, and nonmobilizable plasmids within each lineage. (D) Relationship between median IS/MGE hits and the percentage of predicted conjugative plasmids at the SLV-CC level. Each point represents one lineage. Dashed lines indicate operational visual references (median IS/MGE hits ≥ 10 or conjugative plasmids ≥ 80%) rather than mechanistic thresholds.

Mobilome features varied significantly across internal SLV-CCs, including differences in IS burden and plasmid-associated characteristics (Fig. 5). Across all 10,500 genomes, most genomes contained relatively few IS/MGE hits, with a smaller subset showing markedly higher counts (median 7, interquartile range [IQR] 5 to 11; maximum 383). IS counts differed significantly among SLV-CCs (Kruskal–Wallis test, *P* = 1.92 × 10^−90^; Fig. 5A).

The dominant internal lineage grouping, SLV-CC1, had a median of 7 IS hits per genome (IQR 6 to 11; mean 12.55). Several smaller SLV-CCs showed higher IS counts than SLV-CC1, particularly SLV-CC2, SLV-CC4, SLV-CC5, and SLV-CC14. SLV-CC4 (*n* = 287) had a median of 11 (IQR 8 to 19; mean 19.18), while SLV-CC5 (*n* = 187) and SLV-CC14 (*n* = 127) had mean values of 15.78 and 16.57, respectively. SLV-CC2 (*n* = 81) showed the highest IS burden, with a median of 14 (IQR 9 to 49; mean 39.83), a 95th percentile of 138, and a maximum of 207 (Fig. 5A and Table S19). Pairwise post hoc comparisons are reported in Table S20.

Plasmid replicon diversity also differed across lineages. The number of plasmid replicon types per genome varied significantly among SLV-CCs (Kruskal–Wallis test, *P* = 3.88 × 10^−83^; Fig. 5B). SLV-CC1 had a mean of 2.22 replicon types per genome, whereas higher mean values were observed in SLV-CC108 (2.44) and SLV-CC8 (2.42). In contrast, SLV-CC4 showed a lower mean value (1.19).

Plasmid mobility composition also varied among SLV-CCs (Fig. 5C). Conjugative plasmids were common in SLV-CC108 (93.7%), SLV-CC14 (88.2%), SLV-CC4 (87.1%), and SLV-CC10 (82.7%). In contrast, SLV-CC6 was dominated by mobilizable plasmids (89.1%) with a small proportion of conjugative plasmids (~3%), while SLV-CC8 contained a relatively large fraction of nonmobilizable plasmids (~49%). At the lineage level, the relationship between median IS/MGE hits and the proportion of conjugative plasmids was examined (Fig. 5D). Each point represents one SLV-CC. No significant correlation was observed between these variables (Spearman rho = 0.27, *P* = 0.423).

### Lineage-specific virulome conservation and accessory variation

**Fig. 6.**
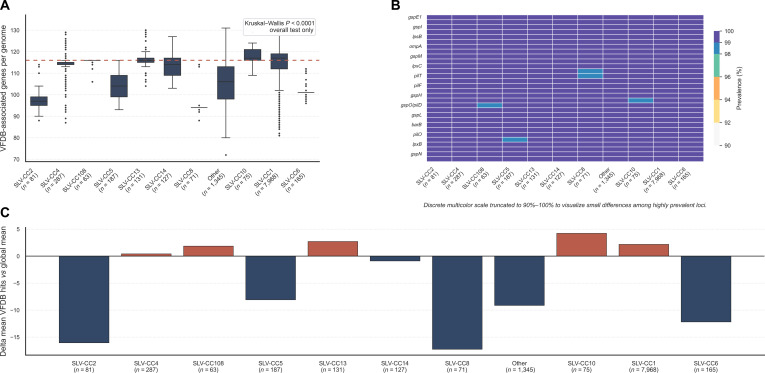
Lineage-structured virulence-associated gene presence across global *Acinetobacter baumannii* lineages. (A) Distribution of Virulence Factor Database (VFDB) hits per genome across Pasteur-based single-locus variant (SLV)-derived clonal complexes (SLV-CCs). Boxplots show the number of virulence-associated genes detected per genome in each lineage (*n* shown below each CC). The dashed red line marks the global median, and the reported *P* value denotes the overall Kruskal–Wallis test. (B) Heatmap of the 30 most prevalent VFDB genes across SLV-CCs. Values represent the percentage of genomes in each lineage carrying the gene. The color scale is truncated to 90% to 100% prevalence to highlight small differences among highly conserved genes. (C) Deviation of mean VFDB gene counts for each lineage relative to the global mean (delta mean VFDB hits *vs* global mean). Positive values represent higher mean counts, while negative values represent lower counts. These profiles summarize gene presence rather than expression or pathogenic activity.

Virulence factor profiling using VFDB showed a largely conserved virulence-associated gene presence profile within the global *A. baumannii* population. Among the 10,500 genomes with VFDB output, the number of detected VFDB-associated genes per genome had a relatively narrow distribution (mean 113.6; median 116; IQR 109 to 118; range 72 to 134), indicating limited variation in total gene presence. Analysis by SLV-CC revealed moderate but statistically significant differences in VFDB gene counts among lineages (Kruskal–Wallis test, *P* = 1.26 × 10^−46^; Fig. 6A). Despite these differences, most SLV-CCs remained close to the global median.

Gene prevalence analysis showed that several virulence-associated loci were highly conserved at the gene presence level (Fig. 6B). Genes detected in >99% of genomes included lipid A and lipopolysaccharide biosynthesis genes (*lpxA*, *lpxC*, *lpxD*, *lpxL*, *lpxM*, and *lpsB*), the outer membrane protein gene *ompA*, phospholipase *plcD*, and multiple genes associated with the type II secretion system (*gspE*, *gspF*, *gspH*, *gspI*, *gspK*, *gspM*, and *gspO/pilD*) and type IV pilus assembly (*pilM*, *pilN*, *pilT*, *pilF*, and *pilH*). These results reflect conservation of gene carriage rather than direct evidence of expression or pathogenic activity.

Comparison of lineage-level averages showed small differences in mean VFDB gene counts relative to the global mean (Fig. 6C). SLV-CC10 and SLV-CC13 showed slightly higher mean counts, whereas SLV-CC2, SLV-CC8, and SLV-CC6 had lower values. Complete VFDB prevalence matrices, functional category summaries, coefficient-of-variation comparisons, and genome-level insertion sequence/virulence factor (IS/VF) integration are provided in Tables S21 to S24. However, the magnitude of these differences was limited across lineages and should be interpreted as modest differences in gene presence rather than direct evidence of altered virulence potential.

### Associations between resistome and mobilome

**Fig. 7.**
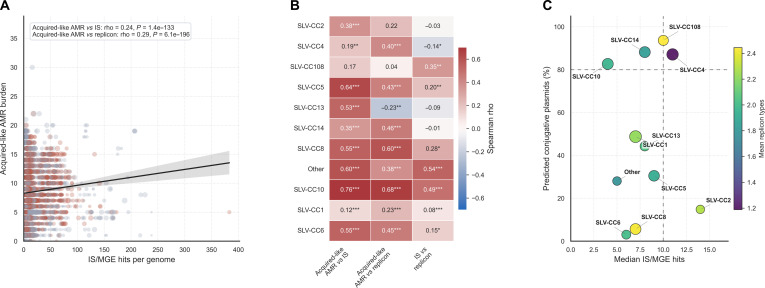
Associations between acquired-like antimicrobial resistance (AMR) burden and mobilome features across global *Acinetobacter baumannii* lineages. (A) Genome-level association between acquired-like AMR burden and insertion sequence (IS)/mobile genetic element (MGE) burden across all 10,500 genomes. Each point represents one genome; point size indicates plasmid replicon type count, and point color indicates predicted conjugative-plasmid signal. Spearman correlation coefficients for acquired-like AMR burden versus IS/MGE burden and plasmid replicon diversity are shown. (B) Spearman correlation coefficients among acquired-like AMR burden, IS/MGE burden, and plasmid replicon diversity calculated separately for each single-locus variant (SLV)-derived clonal complex (SLV-CC). Asterisks indicate statistical significance (*P < 0.05; **P < 0.01; ***P < 0.001). (C) Lineage-level mobilome architecture based on median IS/MGE counts and the proportion of predicted conjugative plasmids. Each point represents an SLV-CC. Bubble size indicates mean acquired-like AMR burden, and color represents mean plasmid replicon diversity. Dashed lines indicate operational thresholds used for visualization (median IS/MGE hits ≥ 10 or conjugative plasmids ≥ 80%).

AMRFinderPlus hit burden showed modest but statistically significant positive correlations with mobilome variables. Total AMRFinderPlus hit burden correlated with insertion sequence (IS/MGE) burden (Spearman rho = 0.259, *P* = 4.26 × 10^−160^) and plasmid replicon diversity (rho = 0.246, *P* = 1.73 × 10^−144^). When the acquired-like burden metric was used instead, the association with IS/MGE burden was rho = 0.237 (*P* = 1.38 × 10^−133^), whereas the association with plasmid replicon diversity was slightly stronger (rho = 0.285, *P* = 6.14 × 10^−196^) (Fig. 7A and Table S26).

Within-lineage analyses using acquired-like AMR burden also showed variable association strength across internal lineage groupings (Fig. 7B). Stronger positive within-lineage correlations between acquired-like burden and IS counts were observed in SLV-CC10 (rho = 0.76), SLV-CC5 (rho = 0.64), the pooled other group (rho = 0.60), SLV-CC8 (rho = 0.55), SLV-CC6 (rho = 0.55), and SLV-CC13 (rho = 0.53), whereas SLV-CC1 displayed only a weak association (rho = 0.12). Associations between acquired-like burden and plasmid replicon diversity also varied across SLV-CCs. These lineage-specific differences should be interpreted cautiously because the SLV-CCs are internally defined analytic groupings of unequal sizes.

These patterns remained detectable in additional sensitivity analyses. After excluding genomes from China and the United States, acquired-like burden remained correlated with IS/MGE burden (rho = 0.247, *P* = 4.60 × 10^−89^) and showed a stronger correlation with plasmid replicon diversity (rho = 0.331, *P* = 5.36 × 10^−162^) (Table S26 and Fig. S10). In lineage-adjusted negative binomial models, both IS burden and plasmid replicon diversity remained positive predictors of acquired-like burden, but the replicon term showed the larger effect size in both the full dataset and the geographic sensitivity analysis (Table S27 and Fig. S10).

Lineage-level mobilome architecture was further compared using median IS counts and the proportion of predicted conjugative plasmids (Fig. 7C). SLV-CC108, SLV-CC4, SLV-CC14, and SLV-CC10 showed high proportions of conjugative plasmids (>80%). In contrast, SLV-CC2 had the highest median IS burden but relatively few conjugative plasmids, whereas SLV-CC6 and SLV-CC8 showed low frequencies of conjugative plasmids.

## Discussion

The AMR landscape in *A. baumannii* is profoundly shaped by its underlying population structure, where clonal inheritance acts as a primary mechanism for maintaining and disseminating key resistance determinants, challenging the notion of resistance being a “horizontally random” phenomenon [36–38]. In this context, the present study aimed to investigate how population structure influences the genomic organization of AMR in *A. baumannii*. Specifically, we analyzed a large global collection of high-quality genomes to characterize lineage-level patterns in the resistome, mobilome, and virulome. By integrating population structure analysis with comparative genomics, this study sought to clarify how resistance determinants are distributed and maintained across dominant evolutionary lineages.

Because population inferences are sensitive to dataset composition, accurate genome curation was prioritized prior to downstream analyses. Restricting the dataset to standardized RefSeq assemblies and applying stringent quality thresholds minimized technical variability that could confound comparative genomic analyses. Exclusion of MAGs and fragmented drafts further reduced analytical artifacts [25,39]. The resulting 10,500 high-quality genomes therefore provide a stable framework for examining lineage-associated resistome and mobilome composition, ensuring that the observed patterns reflect authentic evolutionary signals rather than methodological bias.

The geographic and clinical scope of the dataset further contextualizes these findings. Genomes from 97 countries, with substantial representation from China, the United States, Thailand, and India, reflect previous surveys in which sequencing capacity is concentrated in a limited number of regions. Global collections enriched for Asian isolates have similarly been dominated by high-risk epidemic clones, and regional studies from Thailand and elsewhere in Asia document sustained circulation of carbapenem-resistant lineages in intensive care networks [40–44]. In the present dataset, China and the United States alone contributed 39.6% of all genomes, indicating that country-level counts primarily reflect sequencing infrastructure and surveillance intensity rather than true disease incidence. Collection-year metadata were available for 89.3% of genomes and extended to 2025, further emphasizing that the dataset is cross-sectional and temporally heterogeneous. The predominance of respiratory and bloodstream isolates is consistent with the established role of *A. baumannii* in ventilator-associated pneumonia and invasive hospital infections, while environmental and device-associated isolates reflect its persistence in healthcare settings and capacity to contaminate surfaces and medical equipment, facilitating nosocomial transmission [45–52].

The population structure analysis revealed a highly uneven lineage distribution. In the FastMLST output used here, Pasteur ST assignment was substantially more complete than Oxford numeric ST assignment, and Pasteur ST2 was the dominant lineage. The largest internal SLV cluster (SLV-CC1) contained 7,968 genomes, indicating that much of the dataset is concentrated within a closely related lineage background. This pronounced clonality is consistent with the global expansion of high-risk hospital-associated lineages, but it also means that pooled genome-wide statistics are strongly influenced by sampling imbalance [13,36,37,53–55].

The SLV-CC analysis provides a useful descriptive framework for comparing related sequence types, but these clusters should not be equated with formal international clone labels. Because the CCs were derived internally from SLV relationships within the present dataset, they are best interpreted as analytic lineage groupings rather than standardized epidemiologic entities. This distinction is particularly important when discussing recognized clones such as Pasteur ST2/IC2: the ST-level designation is externally interpretable, whereas the corresponding internal SLV-CC1 label is dataset specific [27,56–59].

Within that constraint, SLV-CC-level summaries remain useful for showing how resistance-, plasmid-, and IS-associated features are distributed across related genomic backgrounds. At the same time, MLST-based clustering cannot fully resolve recombination, deeper phylogenetic splits, or near-identical sampling redundancy, so the lineage structure described here should be interpreted as a pragmatic population-genomic framework rather than a substitute for whole-genome phylogenetic reconstruction [56–58].

The global resistome landscape of *A. baumannii* was clearly lineage structured, but the interpretation of total AMRFinder burden requires caution. Because this metric combines acquired ARGs with intrinsic resistance-associated loci, it does not represent a pure measure of mobilome-mediated ARG accumulation. The near-universal presence of OXA-51-like family genes and other chromosomal loci is consistent with core or lineage-associated background resistance [60], whereas acquired carbapenemases such as OXA-23-like or *bla*_NDM-1_ capture more direct evidence of acquired resistance variation [61–63]. A sensitivity analysis using an acquired-like burden metric reduced the median burden from 17 to 9 hits per genome but preserved positive associations with mobilome features, particularly plasmid replicon diversity. Accordingly, lineage differences in total AMRFinder burden are best viewed as composite resistance-content patterns rather than direct measures of horizontal ARG acquisition [32,60,64].

Mobilome variation among SLV-CCs reflects differences in IS abundance and predicted plasmid mobility profiles among lineages. Some lineages appear to maintain relatively streamlined genomes with limited mobile element expansion, whereas others exhibit pronounced structural plasticity [17,18,65]. This lineage-level variation occurs within the highly open pan-genome of *A. baumannii*, in which ISs and diverse plasmids contribute substantially to genome variability and resistance dissemination [66–73]. Such differences likely influence genome plasticity, although their evolutionary consequences cannot be inferred directly from count-based summaries alone [74,75].

Virulence profiling across more than 10,000 genomes showed that virulence-associated gene presence is comparatively conserved at the species level. Most genomes carried similar numbers of VFDB loci, and the majority of highlighted determinants—particularly those involved in outer membrane structure, secretion, adhesion, and biofilm formation—were nearly universal (Fig. 6 and Tables S21 to S24) [76,77]. Although statistical testing detected differences among SLV-CCs, the magnitude of variation was small compared with the lineage-level heterogeneity observed for mobilome features. These findings support conservation of gene presence, but they should not be interpreted as direct evidence of equivalent expression or pathogenic potential. This interpretation is consistent with previous and recent work emphasizing that *A. baumannii* virulence-associated phenotypes are regulated by processes such as quorum sensing and nucleotide second-messenger signaling, rather than being determined by gene presence alone [78,79].

The association analyses also require cautious interpretation. Genome-wide correlations between AMRFinder burden and mobilome variables were statistically significant but modest in magnitude. In the primary analysis, total AMRFinder burden correlated with IS/MGE burden (rho = 0.259) and plasmid replicon diversity (rho = 0.246). In the acquired-like sensitivity analysis, the corresponding correlations were rho = 0.237 for IS/MGE burden and rho = 0.285 for replicon diversity. After excluding genomes from China and the United States, the acquired-like correlations were rho = 0.247 for IS/MGE burden and rho = 0.331 for replicon diversity (Table S26 and Fig. S10). Lineage-adjusted negative binomial models likewise retained positive effects for both IS burden and replicon diversity, but the replicon term showed the larger effect size in both the full dataset and the geographic sensitivity analysis (Table S27 and Fig. S10). These patterns are consistent with lineage-dependent co-occurrence between resistance-associated and mobilome-associated features, but they do not by themselves demonstrate temporality, causation, ARG genomic context, or specific transfer mechanisms. Similarly, recent transcriptomic analyses of polymyxin and colistin/sulbactam responses in carbapenem-resistant *A. baumannii* highlight that treatment-relevant resistance phenotypes require functional validation beyond genome-content screening alone [80,81].

Several limitations should be considered. First, the dataset is geographically imbalanced, with China and the United States contributing 39.6% of all genomes (Table S28). Second, collection-year metadata were incomplete and no explicit temporal analysis was performed. Third, Mash was used to describe population structure but not as a formal near-duplicate filter before downstream statistics. Fourth, the acquired-like AMR filter used here is a biologically motivated sensitivity analysis rather than a perfect direct label of horizontal acquisition. Fifth, AMRFinder burden, VFDB profiles, and MOB-suite mobility calls are gene-content-based summaries and do not directly resolve ARG genomic context, plasmid localization, resistance-island structure, or functional gene expression [73]. The associations reported here should therefore be interpreted as comparative genomic patterns rather than direct mechanistic proof.

## Data Availability

All genome assemblies analyzed in this study were obtained from the NCBI RefSeq database and are publicly available. There are no restrictions on data availability.
