# Supplementary figures and images for: Lineage-Specific Associations between the Resistome and Mobilome across 10,500 Globally Distributed Acinetobacter baumannii Genomes
Source: Comput Struct Biotechnol J. 2026 Jun 9;35(1):0123. doi: 10.34133/csbj.0123 (PMC13247313; doi:10.34133/csbj.0123)

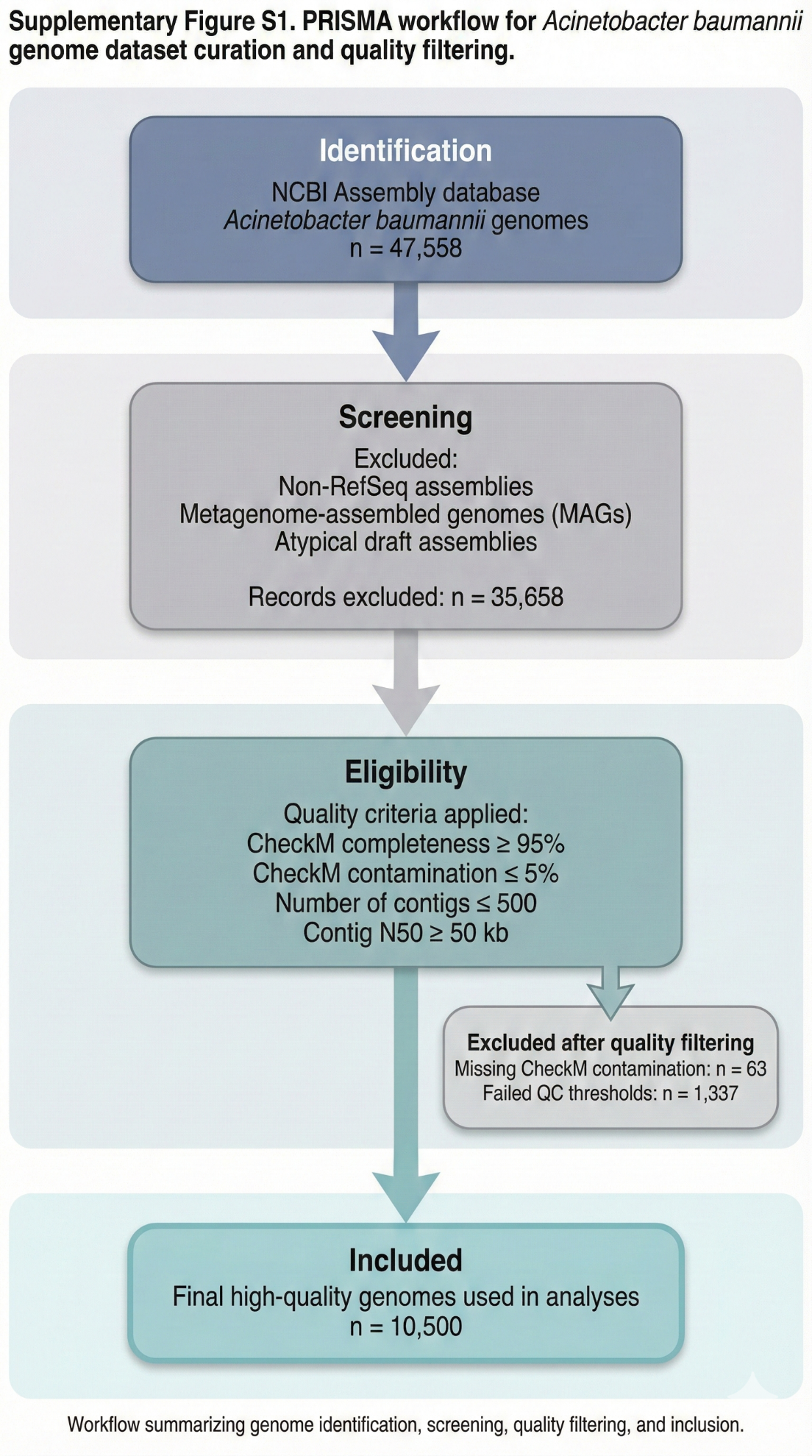

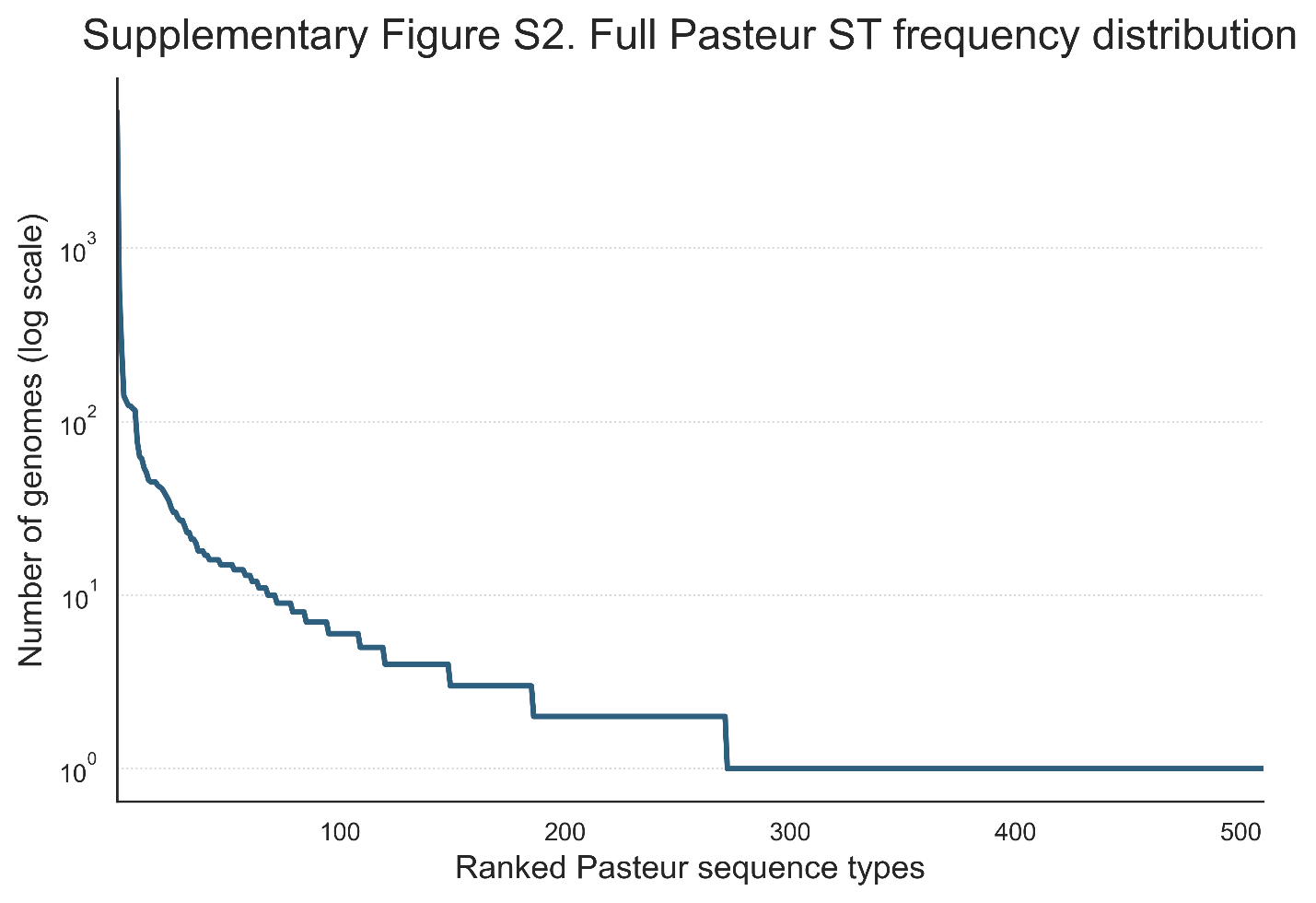

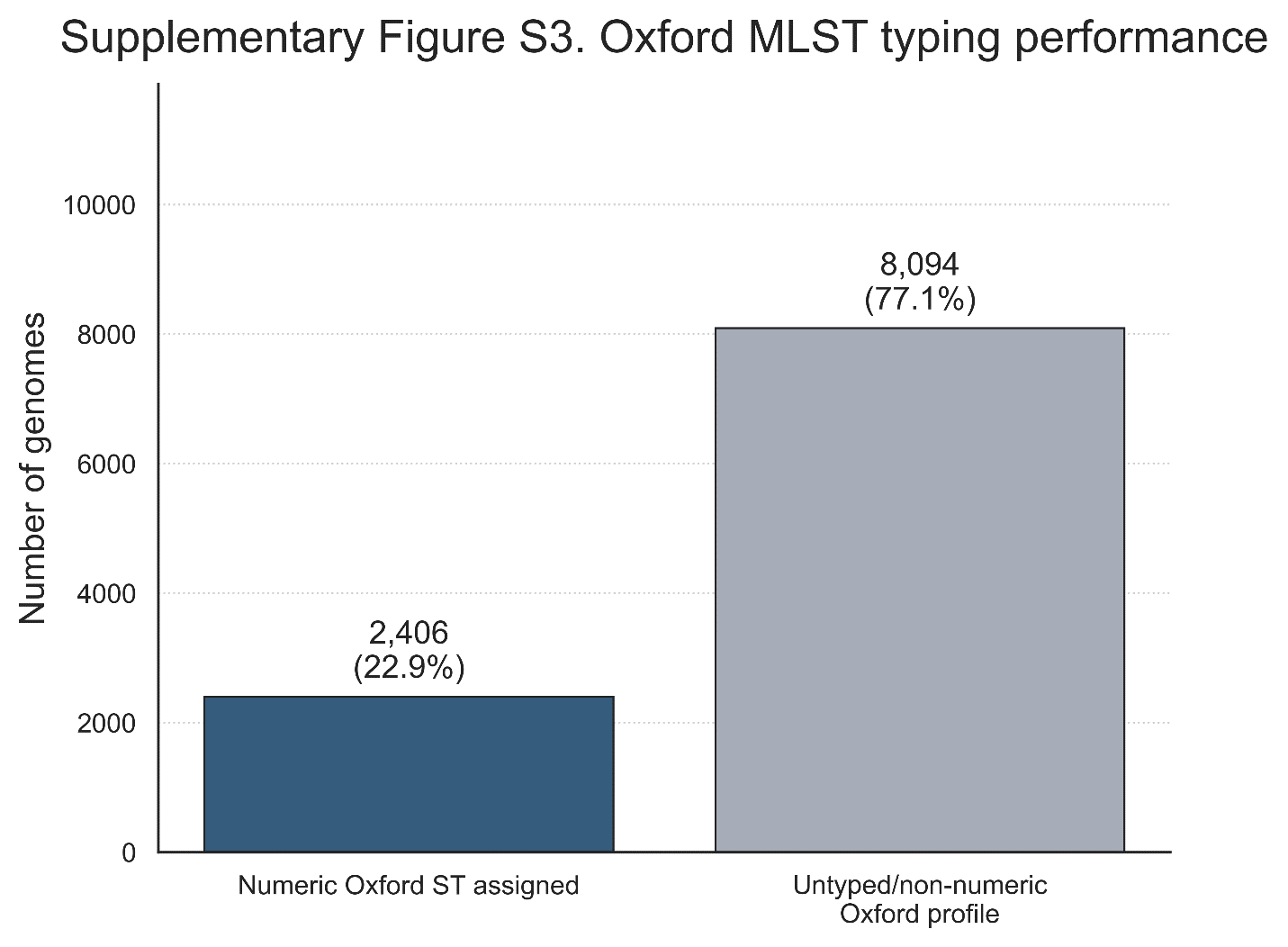

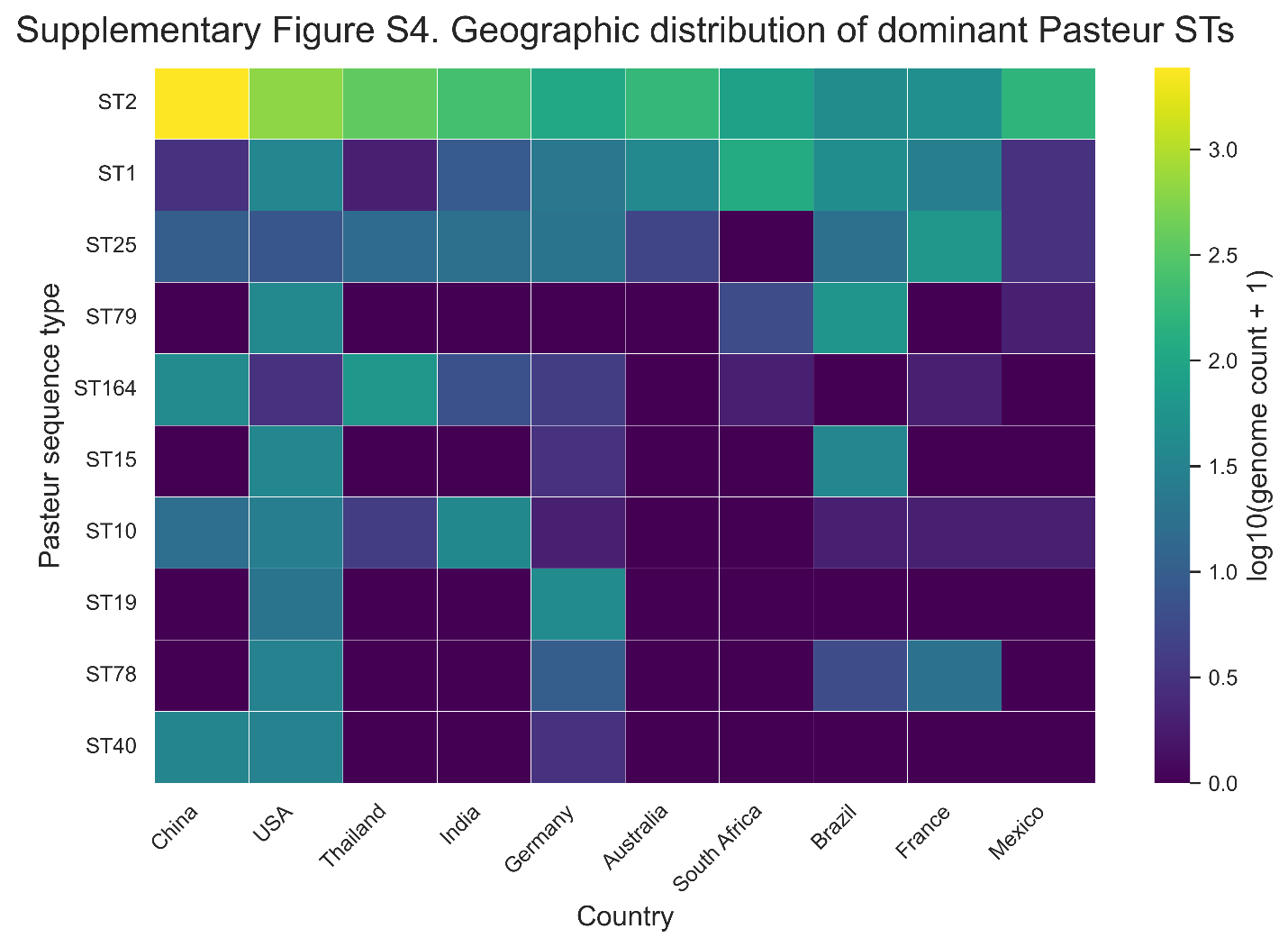


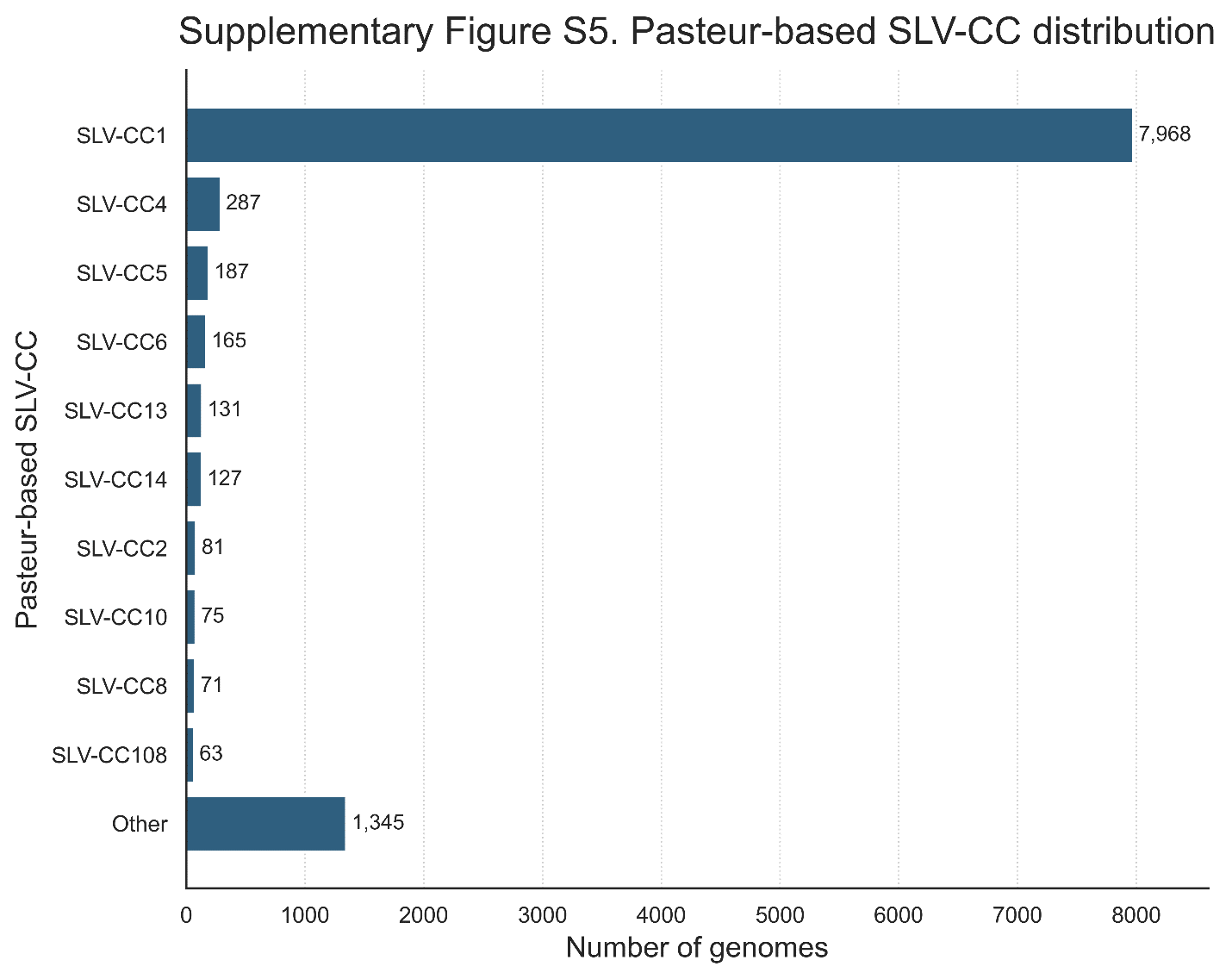

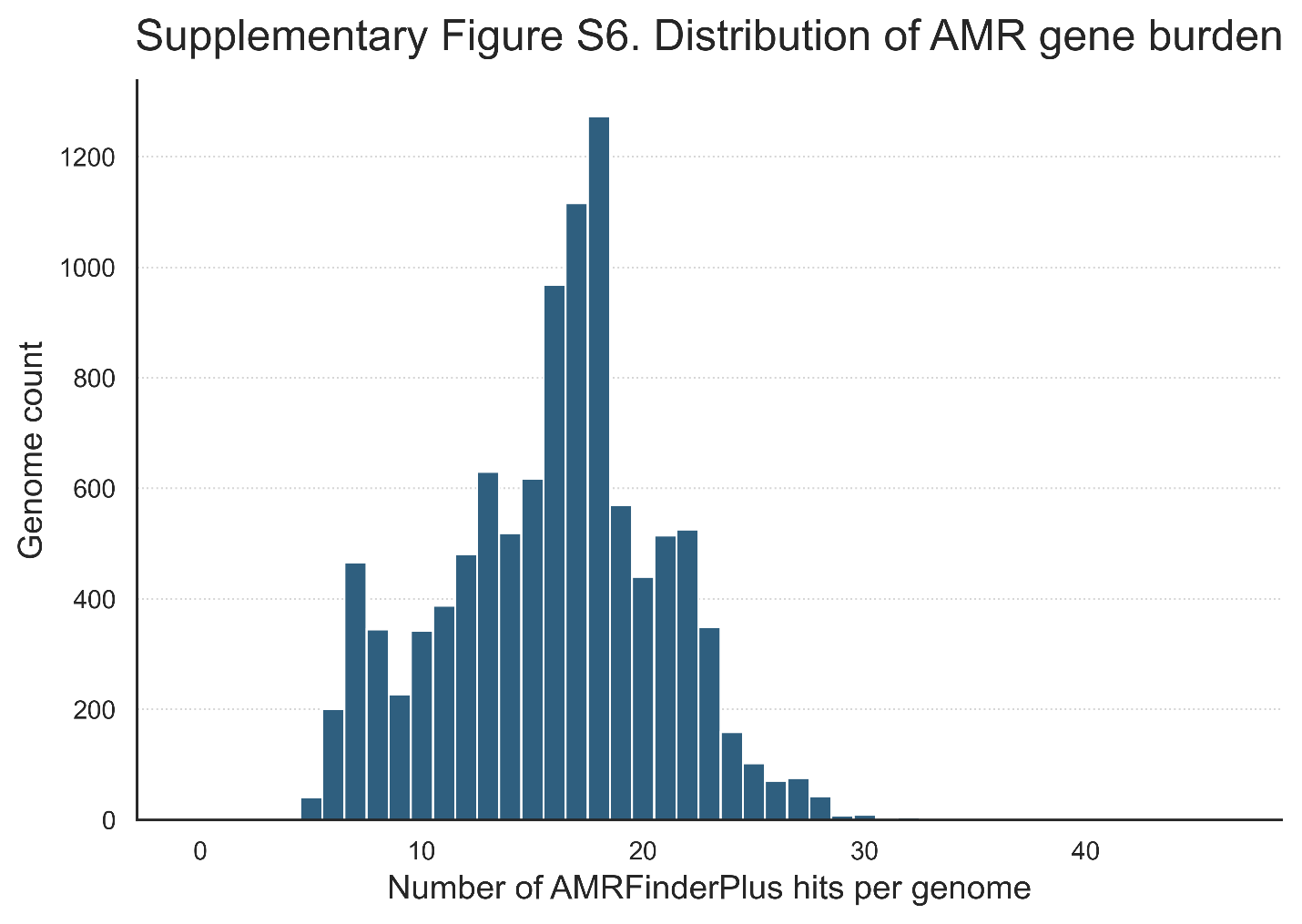

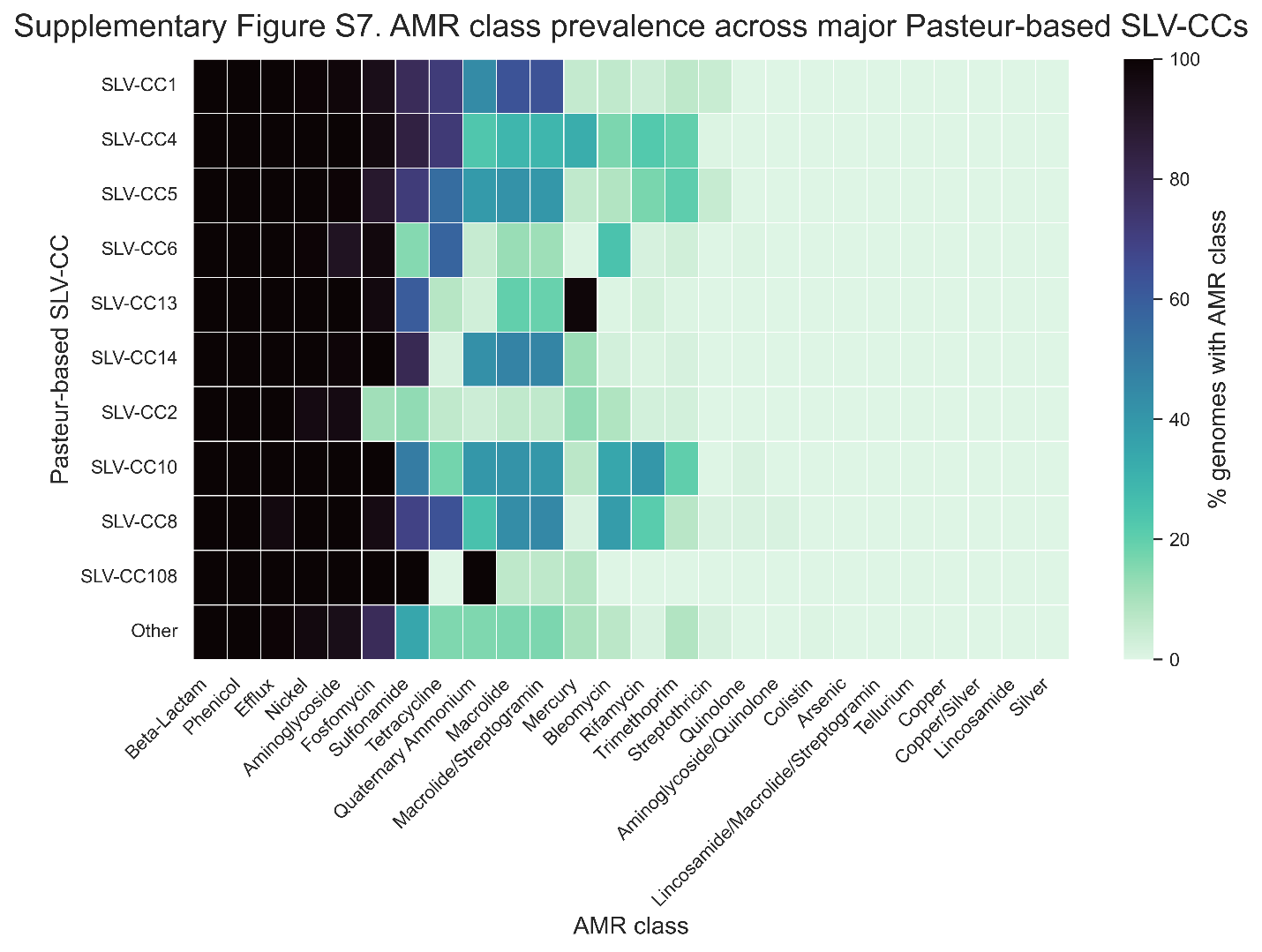

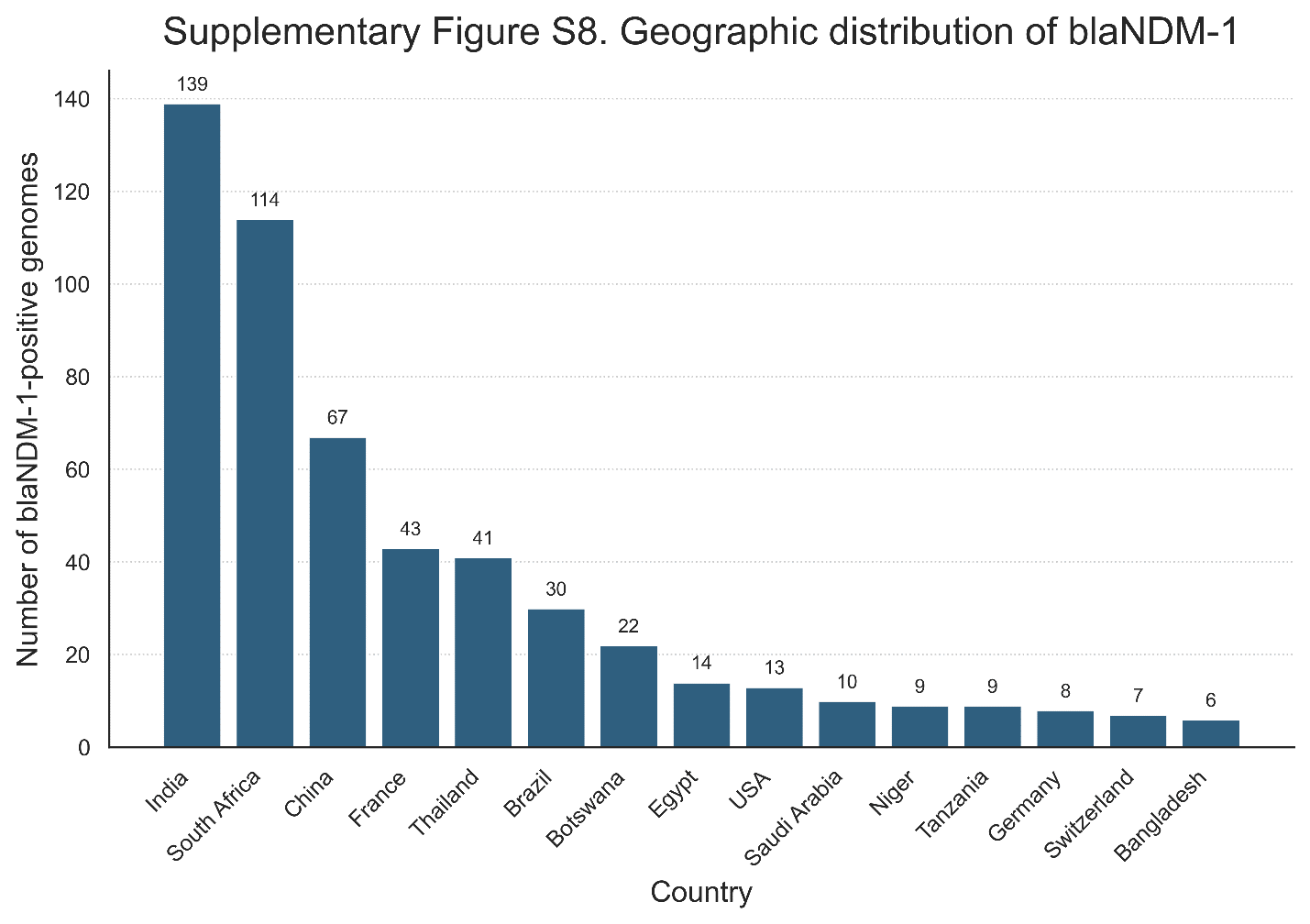

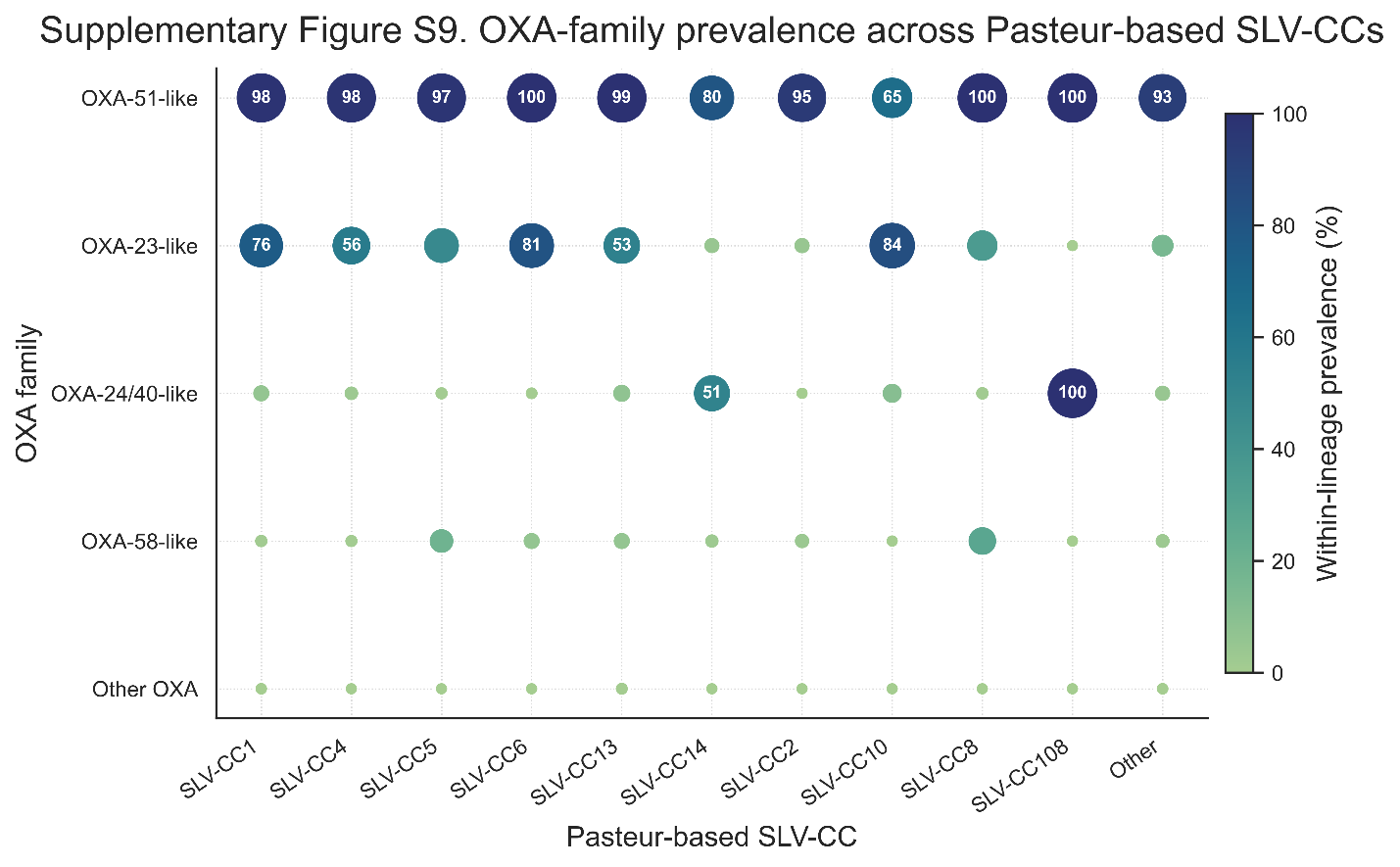

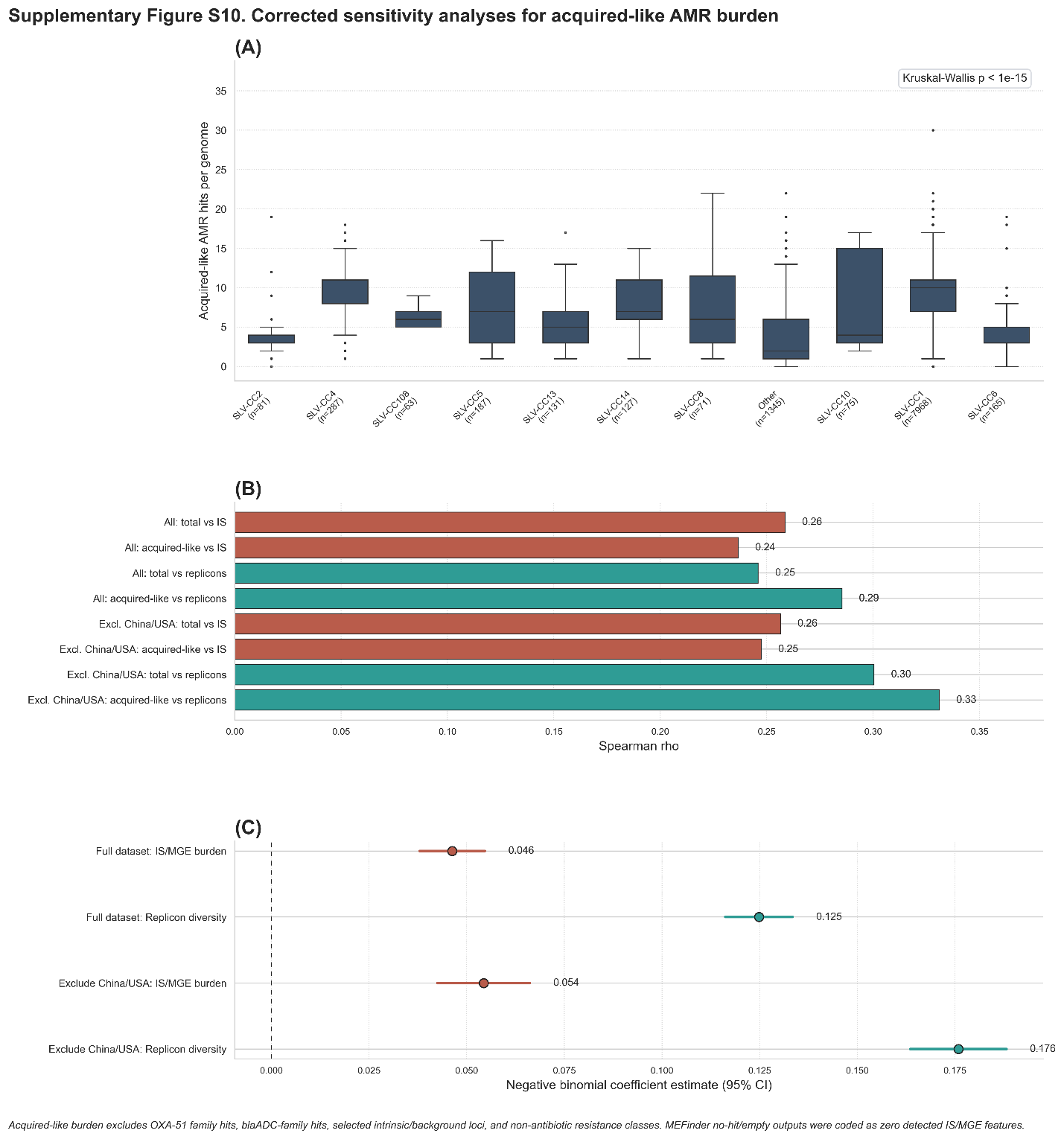

Supplement: Supplementary 1 — Figs. S1 to S10 Tables S1 to S29 [file csbj.0123.f1.zip › Supplementary_Figure.docx]
